# Small Molecules of Marine Origin as Potential Anti-Glioma Agents

**DOI:** 10.3390/molecules26092707

**Published:** 2021-05-05

**Authors:** Ana Alves, Paulo Costa, Madalena Pinto, Domingos Ferreira, Marta Correia-da-Silva

**Affiliations:** 1 UCIBIO, REQUIMTE, Faculty of Pharmacy, University of Porto, Rua de Jorge Viterbo Ferreira 228, 4050-313 Porto, Portugal; anadaniela92@hotmail.com (A.A.); pccosta@ff.up.pt (P.C.); domingos@ff.up.pt (D.F.); 2 Laboratory of Pharmaceutical Technology, Department of Chemical Sciences, Faculty of Pharmacy, University of Porto, Rua de Jorge Viterbo Ferreira 228, 4050-313 Porto, Portugal; 3 Laboratory of Organic and Pharmaceutical Chemistry, Department of Chemical Sciences, Faculty of Pharmacy, University of Porto, Rua de Jorge Viterbo Ferreira 228, 4050-313 Porto, Portugal; madalena@ff.up.pt; 4 Interdisciplinary Center of Marine and Environmental Research (CIIMAR), University of Porto, Terminal de Cruzeiros do Porto de Leixões Avenida General Norton de Matos P, 4450-208 Matosinhos, Portugal

**Keywords:** glioma, marine compound, drug discovery

## Abstract

Marine organisms are able to produce a plethora of small molecules with novel chemical structures and potent biological properties, being a fertile source for discovery of pharmacologically active compounds, already with several marine-derived agents approved as drugs. Glioma is classified by the WHO as the most common and aggressive form of tumor on CNS. Currently, Temozolomide is the only chemotherapeutic option approved by the FDA even though having some limitations. This review presents, for the first time, a comprehensive overview of marine compounds described as anti-glioma agents in the last decade. Nearly fifty compounds were compiled in this document and organized accordingly to their marine sources. Highlights on the mechanism of action and ADME properties were included. Some of these marine compounds could be promising leads for the discovery of new therapeutic alternatives for glioma treatment.

## 1. Introduction

The second leading cause of death in the world is cancer, with a total number of 9.6 million (17% of all deaths) [1]. Glioma is classified by the World Health Organization (WHO) as the most common and aggressive form of central nervous system (CNS) brain tumor and the median overall survival for glioma patients remains around 2 years [2]. WHO classifies gliomas into grades I to IV based on the level of malignancy determined by histopathological criteria, including astrocytic tumors, astrocytoma, anaplastic astrocytoma and glioblastoma (GBM); oligodendrogliomas; ependymomas and mixed gliomas. Gliomas range from grade I, with the least proliferating lesions that can be cured, to grade IV, called cytotoxic malignant neoplasms, which proliferate quickly and invade surrounding healthy tissues [3]. Current treatment is still inadequate due to some tumor related characteristics (such as glioma cells complex composition and diffuse invasiveness) and to blood brain barrier (BBB) selectivity that inhibits drugs from permeating into the brain tumor mass [4,5]. Conventional surgical methods or radiotherapy alone cannot eliminate this kind of tumor, and the relapse is, generally, inevitable [6,7,8]. The conventional used combinatorial therapy is composed firstly by surgery, and then by radiotherapy and chemotherapy [6,8]. However, surgery is the main component of the standard treatment, because reduces the tumor area. Surgical treatment may be followed by radiotherapy and despite a large number of clinical trials already performed, only modest therapeutic results have been achieved [6].

## 2. Treatment of Glioma

**Figure 1:**
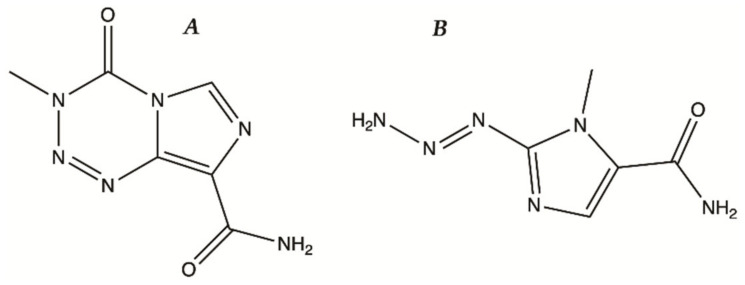
(**A**)—Temozolomide (TMZ); (**B**)—3-methyl-(triazen-1-yl)imidazole-4-carboxamide (MTIC).

Glioma treatment is a challenging task in clinical oncology [9]. Chemotherapy is difficult due to BBB and heterogeneity of this brain cancer [10]. BBB is the main obstacle in routine chemotherapy as it can successfully block many drugs into the bloodstream and hence retard the drug accumulation in the tumor. Additionally, clinical applications are limited by several adverse effects, such as bone marrow suppression, genotoxic, teratogenic and fetotoxic effects. Temozolomide (TMZ, Figure 1A) is the standard care in chemotherapy for glioma, reaching “blockbuster” status in 2010, being the option already approved by Food and Drug Administration (FDA) [11].

TMZ is an imidazotetrazine derivative with an amide group and behaves as a prodrug [12], which spontaneously hydrolyzes at physiological pH to 3-methyl-(triazen-1-yl)imidazole-4-carboxamide (MTIC, Figure 1B). The main mechanism responsible for TMZ cytotoxicity concerns DNA methylation at positions N7 and O6 in guanine, which leads to the failure of the DNA-free repair system to find a complementary base for methylated guanine, resulting in prolonged DNA cuts and consequently blocks of the cell cycle at the G2-M, triggering apoptosis [13]. Some tumor cells express methyl guanine methyl transferase (MGMT) gene and high levels of MGMT activity in tumor cells have been reported to be associated with a poor response to TMZ. MGMT is a critical DNA repair protein that protects tumor cells from alkylating chemotherapeutic agents [14].

Several molecular modifications have been applied to the TMZ scaffold in order to enhance selectivity, targeting activities and improve efficacy via an increased level of BBB penetrance [15,16]. Substitution of the amide group of TMZ for a methylketone increased brain permeability with 69% of the drug entering the CNS compared with 8% for TMZ and produced a more effective compound when tested in mice [5]. In this study, the anticancer activity of two new TMZ analogs replaced at C8 with imidazolyl and methyl imidazole, was evaluated. These results indicate that C8 imidazolyl or methylimidazole confer properties such as greater stability or more efficient delivery [17].

With glioma remaining practically incurable and with an associated short average survival, the search for novel drugs to treat this disease with unique structures and diverse mechanisms of action is a current priority.

**Figure 2:**
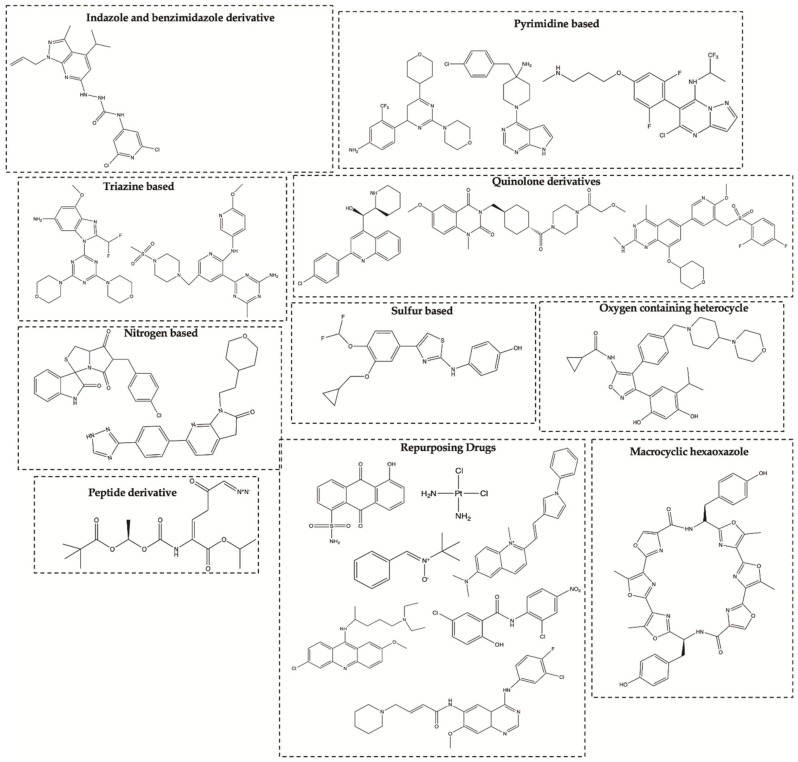
Synthetic compounds that have shown in vivo efficacy against GBM.

Several small synthetic molecules have emerged as potential agents for the treatment of GBM. Nearly a hundred small synthetic molecules with inhibitory activity against GBM cell lines have been described in the last 10 years [18]. Most studies have been carried out in vitro on several glioma cell lines, containing U86MG, U87MG, U118MG, U251MG, U373MG, T98G, SF295 and C6. However, some molecules (Figure 2) have already reached advanced stages of preclinical development with proven efficacy in vivo against GBM [19,20,21,22]. Considering described targets several protein kinases have been referred such as phosphoinositide 3-kinase (PI3K), dual-specificity tyrosine-regulated kinases (DYRK), pyruvate dehydrogenase kinase 1 (PDK1), casein kinase II (CK2), c-Src, protein kinase B (Akt), focal adhesion kinase (FAK) and epidermal growth factor receptor (EGFR) [23,24,25,26]. In addition, many cellular pathways, enzymes and processes have also been investigated, such as G-quadruplexes, histone deacetylases (HDACs), heat shock protein 90 (HSP90), microtubules, via NF-kB, p53, among others [23,24,25,26,27,28].

## 3. Marine Organisms as Sources of Anti-Glioma Compounds

Interest in the oceans has grown in recent decades due to the enlargement of discovery in quantity and diversity of marine products with interesting biological/pharmacological activities [29,30,31]. Over the past fifty years, progressive improvements have been made in the exploration of new marine habitats, leading to the isolation of thousands of unique marine natural products [30] for industrial development, such as pharmaceuticals, food ingredients, cosmetics, drug delivery systems (DDS) and industrial enzymes [31,32,33]. Species such as algae, sponges and corals are in constant competition because they are at high risk of predation, so these species have developed chemical defense mechanisms based on the synthesis of toxic secondary metabolites [34].

In the last decade, FDA has approved a macrolide (Halaven^®^, 2010) derived from a sponge (*Halichondria okadai*) for metastatic breast cancer; an antibody-drug conjugate (Adcetris^®^, 2011) isolated from a sea hare (*Dolabella auricularia*) for use in Hodgkin’s lymphoma and anaplastic large cell lymphoma, and an alkaloid (isolated from a tunicate) (Yondelis^®^, 2015) for ovarian cancer and soft tissue sarcoma [35,36]. Therefore, the search for antitumor compounds derived from the sea has proved to be worthy.

The purpose of this review is to bring together marine compounds described with anti-glioma effects in the last decade. Most of the anti-glioma compounds discovered were isolated from anemones, seaweed (brown and green), bacteria, sponges, invertebrates, fungi and corals and will be following described accordingly to these sources.

### 3.1. Marine Anemone

**Figure 3:**
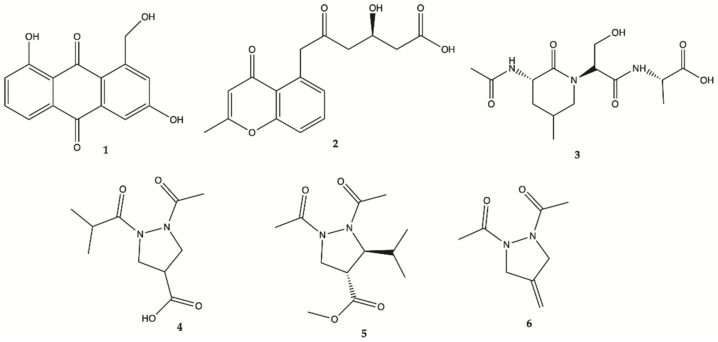
Compounds **1**–**6** isolated from the culture of *Streptomyces* sp. *ZZ406* [40].

An extract prepared from the culture of a sea anemone-derived actinomycete *Streptomyces* sp. ZZ406 [33] was found to have activity in inhibiting the proliferation of glioma cells and reducing the production of lactate in glioma cells [37,38,39]. Chemical investigation of this crude active extract led to the isolation and identification of compounds **1**–**4** (Figure 3) [40]. The structure of compound **1** was elucidated as 1-hydroxymethyl-8-hydroxy-anthraquinone-3-carboxylic acid, a new anthraquinone [40]. These new compounds **1**–**4** were also tested for their activity in inhibiting the proliferation of human glioma U87MG, U251 and SHG44 cells by sulforhodamine B (SRB) assay. Doxorubicin (DOX), a chemotherapeutic drug, was used as a positive control. It has been found that **1** had potent activity against different glioma cells with IC_50_ values in a range of 4.7 to 8.1 μM, and good stability. Compound **2**, a chromone, showed IC_50_ values of 21.6–25.8 μM. Unfortunately, the new peptide **3** and new pyrazolidine derivative **4** were inactive. DOX had an antiproliferative activity with IC_50_ values of 1.9–9.6 μM. Pyrazolidenes 5 and 6 were also assayed for their activity against glioma. The results showed that both pyrazolidines **5** and **6** also had anti-glioma activity with IC_50_ values of 0.5 to 3.0 μM for **5** and 10.4 to 36.3 μM for **6**. The cytotoxicity (CC_50_) of the two active compounds **1** and **2** towards normal human astrocytes (HA) was also evaluated and an IC_50_ higher than 100 μM was found showing high selectivity index (>12.3 to 21.3). They were also analyzed for their effects on the expression levels of important tumor glycolytic (regulatory) enzymes—hexokinase (HK2), 6-phosphofructo-2-kinase/2,6-bisphosphatase 3 (PFKFB3), pyruvate kinase M2 (PKM2) and lactate dehydrogenase (LDH5), highly expressed in U87MG 27 cells. Compounds **1** and **2** clearly reduced the levels of HK2, PFKFB3, PKM2 and LDH5 expression [40].

### 3.2. Seaweed

Seaweeds (also known as macroalgae) comprise a very large number of species of marine algae that are macroscopic and multicellular. This designation “seaweeds” includes Rhodophyta (red), Phaeophyta (brown) and Chlorophyta (green) macroalgae. Only one compound isolated from red algae was described as active in glioma cells and its mechanism of action is thought to be related to the inactivation of the AKt pathway. Regarding brown algae, at least three compounds from different chemical classes, phorotannin, saccharide and carotenoids, were described. It should also be noted that these compounds have different mechanisms of action: (i) eckol was shown to suppress PI3K-AKt activity and inhibit the Ras-Raf-1 pathway, (ii) fucoidan was reported to stimulate differentiation of osteoblasts via c-Jun N-terminal kinase (JNK), (iii) fucoxanthin showed preventive effects through different mechanisms of action. A large number of studies [41,42,43,44] have shown that these different mechanisms of anticancer action include anti-proliferation, suppression of angiogenesis, cell cycle arrest, apoptosis induction and antidrug potential. Regarding green algae, four compounds from the same chemical class, Bafilomycins, were described with antiproliferative activity on human glioma cells [43].

#### 3.2.1. Red Seaweeds 

**Figure 4:**
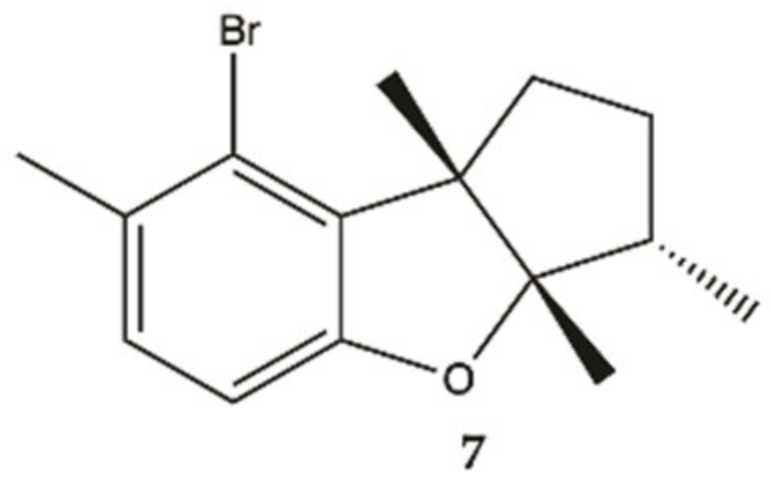
Sesquiterpene **7** isolated from marine red seaweed [44].

Aplysin (**7**, Figure 4), is a bromo sesquiterpene isolated from *Laurencia tristicha*. This compound was found to reduce the number of invasive glioma cells U87MG and U251MG, as well as primary glioma cells, in a dose-dependent manner [44]. Furthermore, the Akt pathway was inactivated by aplysin, and reactivation of the Akt pathway rescued its inhibitory effect on proteins associated with invasion and the invasiveness of U87MG cells. The Akt pathway is related to cell proliferation, protein synthesis, survival and motility and increased expression of these proteins has been associated with tumors with a worse prognosis. No cytotoxicity for normal cells (normal neuronal cell line, HCN2, normal liver cell line, L-02, normal endothelial cell line, HUV-EC-C and normal lung fibroblast cell line, MRC-5) was observed in the presence of aplysin (400 μg/mL) [44].

#### 3.2.2. Brown Seaweeds

**Figure 5:**
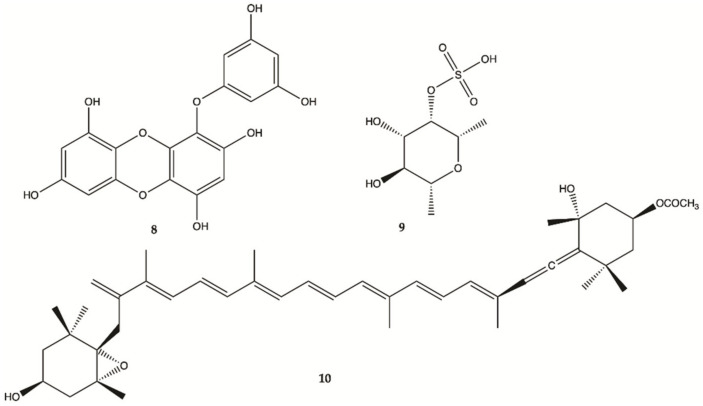
Compounds **8**–**10** isolated from brown seaweed [41,45].

Eckol (**8**, Figure 5), is a phlorotannin component of some brown algae, including *Ecklonia cava* (Laminariaceae), and was shown to protect cells from H_2_O_2_-induced oxidative stress via radical quenching and catalase activation [41]. In this study, phosphoinositide 3-kinase (PI3K)-Akt and Ras-Raf-1-Erk pathways are previously found to be activated in cancer stem-like cells whether Eckol inhibits these signaling pathways. Treatment with eckol caused a marked suppression of PI3K-Akt activities, and completely inhibited Ras-Raf-1 interaction and Raf-1 and Erk activations in sphere-forming glioma stem-like cells. It was hypothesized that eckol may enhance the sensitivity of glioma stem-like cells to anticancer treatments such as ionizing radiation or chemical drugs via inhibition of PI3K-Akt and Ras-Raf-1-Erk pathways.

Fucoidan (**9**, Figure 5), a sulfated saccharide isolated from *Fucus vesiculosus*, inhibited both glioma cell-induced and monocyte-induced angiogenesis in vitro. The effects of fucoidan on T98G-induced or THP1-induced angiogenesis were also evaluated by tube formation assay using conditioned medium of fucoidan-treated cells. After 24 h, T98G and THP1 cell-induced tube formation was inhibited by fucoidan (100 mg/mL) [45]. No apoptosis was observed in human lens epithelial cells (SRA) indicating the specific action of fucoxanthin against carcinogenic cells. Fucoxanthin (**10**, Figure 5) is one of the most abundant carotenoids and contributes to more than 10% of the estimated total production of carotenoids in nature, especially in the marine environment [46]. Fucoxanthin is a pigment that, along with chlorophylls and β-carotene, is widely distributed in brown algae and diatoms. Fucoxanthin has been described with different mechanisms of action including anti-proliferation cell cycle arrest, apoptosis induction, suppression of angiogenesis among others [42].

#### 3.2.3. Green Seaweeds

**Figure 6:**
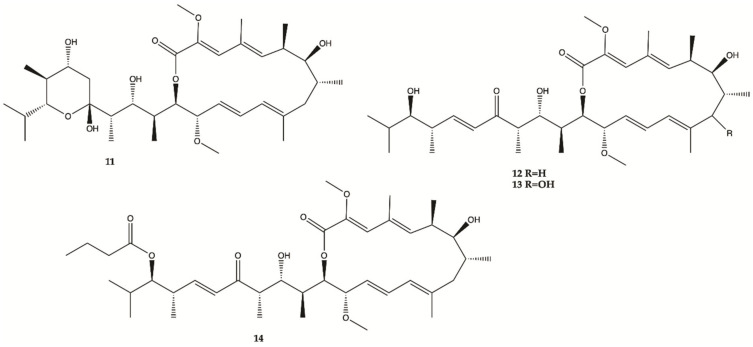
Bafilomycins **11**–**14** isolated from marine green algae *Ulva pertusa* [43].

A methanol extract obtained from a culture of an actinomycete *Streptomyces* sp. HZP-2216E isolated from marine green algae *Ulva pertusa* significantly inhibit proliferation of human glioma cells. Therefore, 4 Bafilomycins were isolated from this extract (**11**–**14**, Figure 6), and assayed for their activity against proliferation of human glioma U87-MG, U251 and SHG44 cells as well as rat glioma C6 cells by SRB assay. Bafilomycin A1 (**11**), bafilomycin D (**12**), 9-hydroxybafilomycin D (**13**) and 23-*O*-butyrylbafilomycin D (**14**) showed potent activity in the suppressing of the proliferation of the four tested glioma cell lines with IC_50_ values in a range from 0.35 to 2.95 μM. The control DOX had similar activity with IC_50_ values of 0.48 to 1.76 μM [43]. Unfortunately, these compounds also showed potent activity against HA with IC_50_ values of 0.22 μM for **11**, 0.42 μM for **12**, 0.06 μM for **13**, and 0.14 μM for **14**.

### 3.3. Marine Bacteria

Marine bacteria are organisms from which various compounds from different chemical families have been isolated, such as Actinomycins, Fradimicins and Streptoglutarimides. Regarding Actinomycins, three compounds were isolated, showing significant inhibition of the growth and proliferation of different glioma cell lines. Interestingly, it was discovered that Actinomycins D (**15**) negatively regulate various metabolic enzymes of the glioma from different metabolic pathways (glycolysis, glutaminolysis and lipogenesis). In this study, Actinomycins D (**15**) and V (**16**) showed values of IC_50_ of 1.01–10.06 μM and 0.42 to 1.80 μM, respectively. From the Fradimicins, two compounds were identified and isolated showing potent activity against glioma cells, but Fradimicin (**18**) showed an IC_50_ of 0.47 μM and induced apoptosis and necrosis of HCT-15, SW620, C6 cells and blocked HCT-15 cells in the phase G0/G1. Finally, from Streptoglutarimides, only two compounds were also isolated, with compound **21** showing an IC_50_ value of 0.05−0.22 μM and revealing more potent antiproliferative activity against glioma cells than compound **20**.

**Figure 7:**
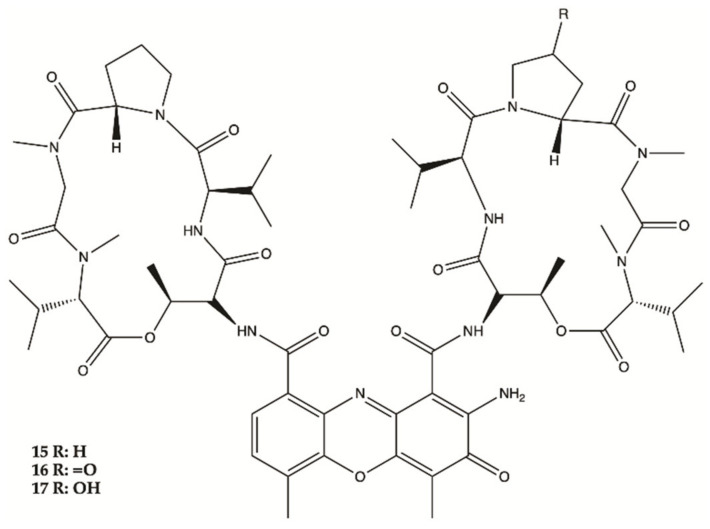
Actinomycins **15**–**17** isolated from sea squirts [37].

Three Actinomycins D (**15**), V (**16**) and X0β (**17**) (Figure 7) produced by a *Streptomyces* sp. strain, ZZ338, isolated from sea squirts, were tested for their activity against the proliferation of human glioma U251 and SHG44 cells and rat glioma C6. The data obtained from this study indicated that the referred three Actinomycins had potent activity against the proliferation of the three tested tumor cell lines, with IC_50_ values from 1.01 to 10.06 nM for actinomycin D (**15**), 0.42 to 1.80 nM for actinomycin V (**16**), 3.26 to 25.18 nM for actinomycin X0β (**17**), while the control DOX showed activity with IC_50_ values in a range from 0.70 to 9.61 μM. In this study, actinomycin D (**15**) was found to significantly downregulate the expression levels of several glioma metabolic enzymes, including HK2 and PKM2 from glycolysis, GLS from glutaminolysis and FASN from lipogenesis [37].

**Figure 8:**
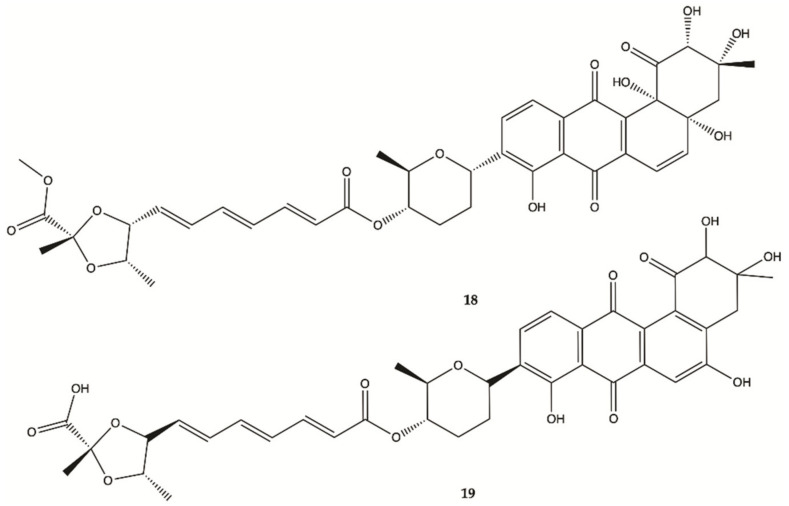
Fradimicins **18** and **19** isolated from *Streptomyces fradiae* PTZ0025 [47].

Two new Fradimicins (**18** and **19**, Figure 8) were isolated and identified from marine *Streptomyces fradiae* PTZ0025. Fradimicins **18** and **19** showed significantly inhibit cell growth of rat C6 glioma cells. Fradimycin **19** induced apoptosis and necrosis of C6 cells [47].

**Figure 9:**
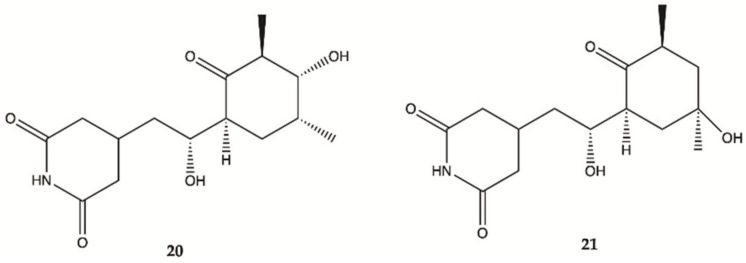
Streptoglutarimides **20** and **21** isolated from Actinomycete Derivates *Streptomyces* sp. ZZ741 [48].

Streptoglutarimides **20** and **21** (Figure 9) were isolated from an actinomycete, *Streptomyces* sp. ZZ741. They showed potent antiproliferative activity against human glioma U87MG and U251 cells with IC_50_ values of 1.5−3.8 μM for **19** and 0.05−0.22 μM for **20** [48].

### 3.4. Marine Invertebrates

One anthraquinone and three alkaloids were isolated from marine invertebrates and studied in glioma cell lines as follows.

**Figure 10:**
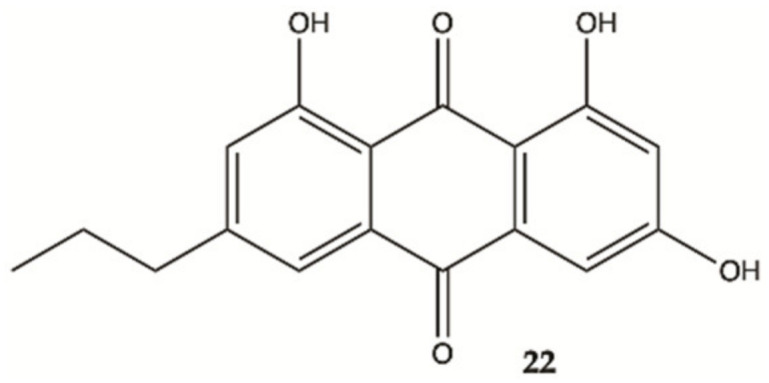
Antraquinone **22** isolated from the marine *Echinoderm Comanthus* sp [49].

An anthraquinone (**22**, Figure 10), isolated from the marine echinoderm *Comanthus* sp., showed prominent toxic effects to C6 glioma cells up to 50 μM (IC_50_ value of 23.2 μM). A significant increase in caspase 3/7 activity was found in C6 glioma cells showing apoptotic cell death. Incubation of C6 cells with 25 μM of compound **22** resulted in an increase in LDH activity [49].

**Figure 11:**
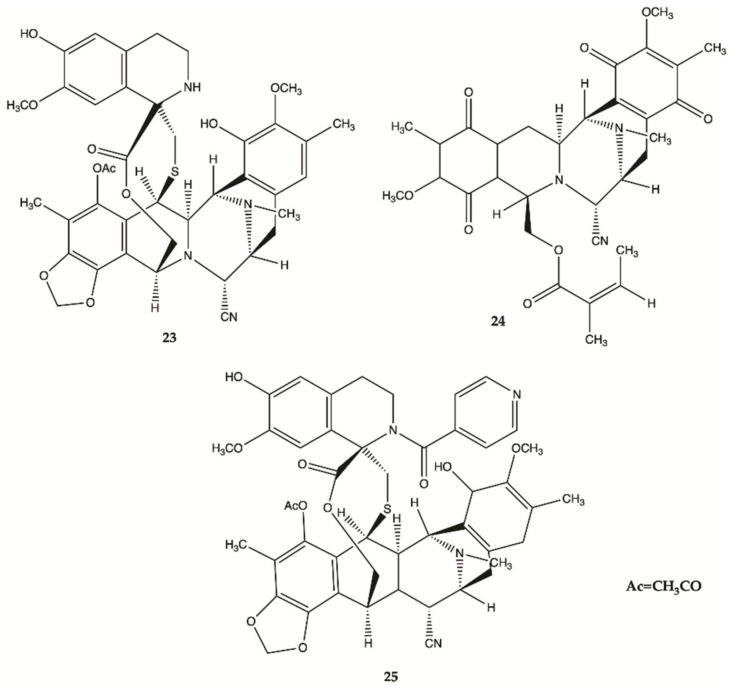
Alkaloids **23**–**25** isolated from Thai marine invertebrates [50].

More recently, a series of 1,2,3,4-tetrahydroisoquinoline alkaloids **23**–**25** were obtained from Carribean Tunicate *Ecteinascidia tubinata* [50] with potent anti-cancer activities, including ecteinascidin-770 (**23**, ET-770), renieramycin M (**24**, RM), as well as a 2′-N-4”-pyridinecarbonyl derivative of ET-770 (**25**) (Figure 11). These compounds induced apoptosis of U373MG cells at nanomolar concentrations. The molecular pathways responsible for cytotoxic effects of these compounds on the human glioma cell line U373MG were characterized. The ErbB signaling pathway (EGFR) is composed of FAK/protein tyrosine kinase 2 (PTK2), Akt3, and glycogen synthase kinase 3 beta (GSK3B), serving as key molecules involved in cell movement and nervous system development. Compounds **23**–**25** showed a significant relationship with the cell cycle pathway, where cell division cycle 25A (CDC25A) acts as a central molecule. Finally, we found that a specific inhibitor of GSK3B induced apoptosis of U373MG cells, supporting an anti-apoptotic role of GSK3B. These observations indicate that molecular network analysis is a useful approach not only to characterize the pathways relevant to the glioma but also to identify effective targets for network-based drugs [50]. These alkaloids induced apoptosis of glioma cells through shared molecular mechanisms involving various pathways and targets, that play a key role in the survival and invasion of glioma cells [50].

### 3.5. Marine Sponges

Different chemical classes of compounds with anti-glioma activity, namely alkaloides, plakortide, sesquiterpenes and sphingosinas, were isolated from marine sponges.

**Figure 12:**
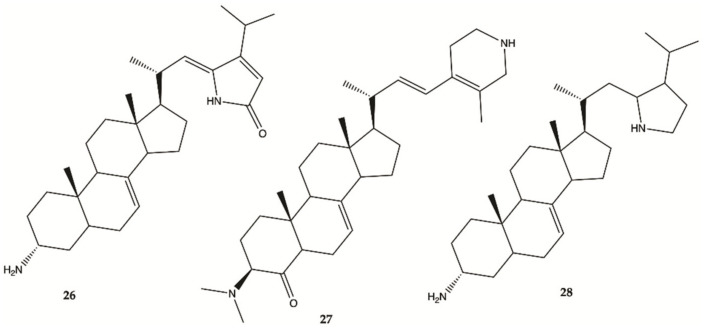
Alkaloids **26**–**28** isolated from Marine Sponge *Corticium* sp. [51].

Steroidal alkaloids **26**–**28** (Figure 12) were isolated from a sample of *Corticium* sp. collected from Porth Havannah, Vanuatu, South Pacific. Cytotoxicity was evaluated in a rat glioma C6 and murine monocyte/macrophages (RAW 264) cell lines. Compound **28** to be the most active with an IC_50_ value of 1.4 μg/mL against rat glioma C6, whereas compound **26** was without effect RAW 264 cell lines, but the compounds **27** (IC_50_ 61.0 μg/mL) and **28** (IC_50_ 16.2 μg/mL) were shown to be cytotoxic against RAW 264 [51].

**Figure 13:**
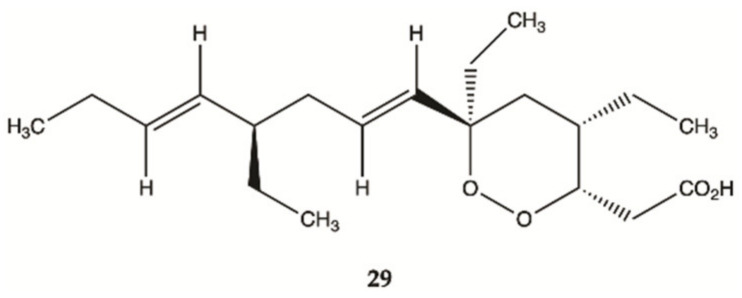
Plakortide **29** isolated from Marine Sponge *Plakortis halichondroides* [52].

Compound **29** (Figure 13) isolated from sponges from the Caribbean Sea was tested for its anticancer effects on human malignant glioma cells U87MG and U373MG. Compound **29** had an IC_50_ value of 4.0 μmol/L on U373MG cell line [52].

**Figure 14:**
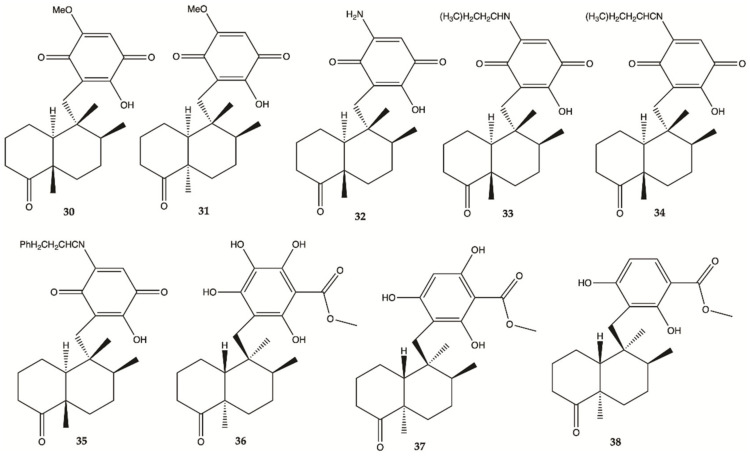
Sesquiterpenes **30**–**38** isolated from extracts of the Hawaiian marine sponge *Dactylospongia elegans* [53].

Sesquiterpene **30**–**38** (Figure 14) were isolated from the extracts of the Hawaiian marine sponge *Dactylospongia elegans*. These nine compounds showed strong to moderate cytotoxicity (IC_50_ values of 2.4–19.4 μM) against the human glioma cancer cell line, U251MG (Table 1) [53].

**Figure 15:**
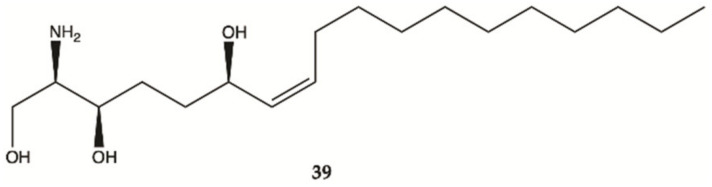
Sphingosine **39** isolated from a marine sponge *Haliclona tubifera* [54].

Compound **39** was isolated from a marine sponge *Haliclona tubifera*, which is abundant species on the southern Brazilian coast (Figure 15). This compound showed a cytotoxic effect in human glioma cell lines (U87MG), with a value lower than IC_50_ < 15 μg/mL [54].

Overall, marine sponges from Brazilian, Hawaiian, Caribbean and South Pacific coasts were explored for their ability to produce secondary metabolites with anti-glioma activity. Concerning alkaloids, compound **28** was the most active. Plakortide (compound **29**) has a core potent and effective against glioma cell lines. Considering sesquiterpenes, compounds **32** and **36** showed the highest potency. The only sphingosine (compound **39**) isolated from marine sponges showed high activity against glioma cells.

### 3.6. Marine Corals

Seven compounds extracted from marine corals showed activity against human malignant glioma cells.

**Figure 16:**
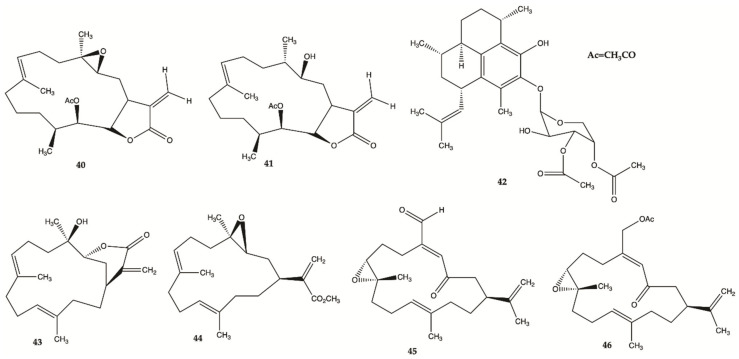
Polyketides **40**–**46** isolated from *Caribbean Octocoral Eunicea succinea* [52].

Compounds **40**–**46** (Figure 16) are derived from specimens of Caribbean Gorgonian Octocoral *Eunicea succinea*. These compounds were encoded and examined blindly for their relative cytotoxicity against human malignant glioma cells U87MG and U373MG. The cells were treated with each compound from 0 to 100 mol/L for 72 h and viability was determined using the 3-(4,5-dimethylthiazol-2-yl)-2,5-diphenyltetrazolium bromide assay. Compound **40** was stable for at least several months and was the most potent compound exhibiting IC_50_ values of 5.1 mol/L for U87MG cells and 6.9 mol/L for U373MG cells. This IC_50_ range is compatible with that of cisplatin, which is in the range of 5 to 10 mol/L for these types of cells. These results lead to focus on mechanism of action of compound **40** [52]. Compound **40** induced interruption of the G2-M cell cycle and apoptosis via the mitochondrial pathway. This compound was found to increase the phosphorylated JNK by >50% in both U87MG and U373MG cells. A specific JNK inhibitor, SP600125, inhibited apoptosis, confirming the involvement of the JNK pathway in cell death by compound-induced apoptosis. In addition, seven days of daily intratumor injections of compound **40** significantly suppressed the growth of malignant glioma xenografts [52].

### 3.7. Marine Fungi

In 2018, one alkaloid with antiproliferative activity against human glioma cells was isolated from a marine fungus strain ZZ380 isolated from a wild crab [55].

**Figure 17:**
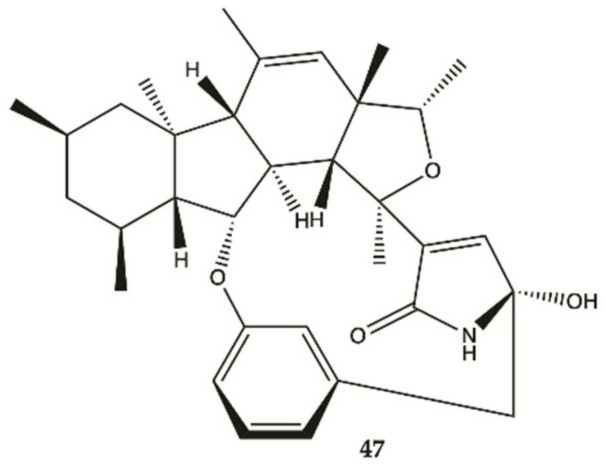
Alkaloid **47** isolated from marine fungus strain ZZ380 [55].

The proliferation of glioma U87MG, U251, SHG44 and C6 cells in the presence of penicipyrroether alkaloid (**47**, Figure 17) was assayed by SRB assay. Compound **47** showed potent activity in inhibiting the proliferation of different glioma cells with IC_50_ values of 1.64 e 5.50 μM for U87MG and U251 cells, respectively [55].

### 3.8. Cucumbers

**Figure 18:**
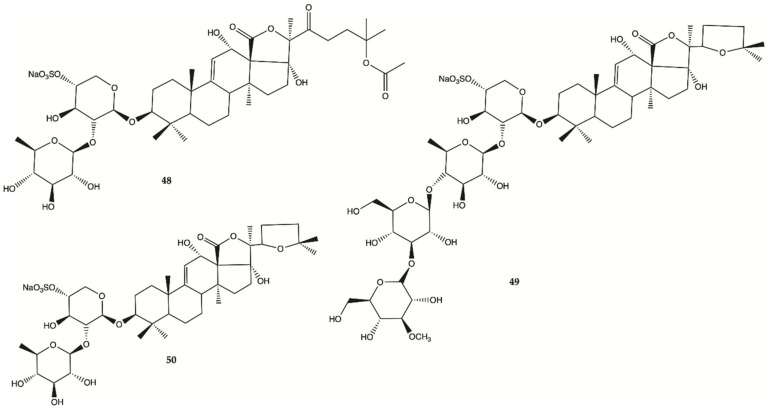
Sulfated saponins **48–50** isolated from *Cucumber Holothuria moebii* [56].

Sulfated saponins were isolated from sea cucumber *Holothuria moebii* Ludwig, a species of sea cucumbers of the Holothuriidae family. For the first time, sulfated saponins **48–50** (Figure 18) were shown to have potent activity in suppressing the proliferation of glioma cells (C6 glioma cells, U87MG, U251 and SHG44 cells). This study also uncovered that sulfated saponin **48** could have a unique antitumor mechanism by selectively targeting multiple glioma metabolic regulators of glycolysis and glutaminolysis, hexokinase 2 (HK2), 6-phosphofructo-2-kinase/2,6-bisphosphatase 3 (PFKFB3), pyruvate kinase (PKM2) and glutaminase (GLS). It has been revealed that these metabolic regulators are related to the tumorigenesis of gliomas [56]. Saponin **48** had no significant effect on the expression levels of HK2, PFKFB3, PKM2 and GLS in HA.

### 3.9. Crustaceans and Fishes

**Figure 19:**
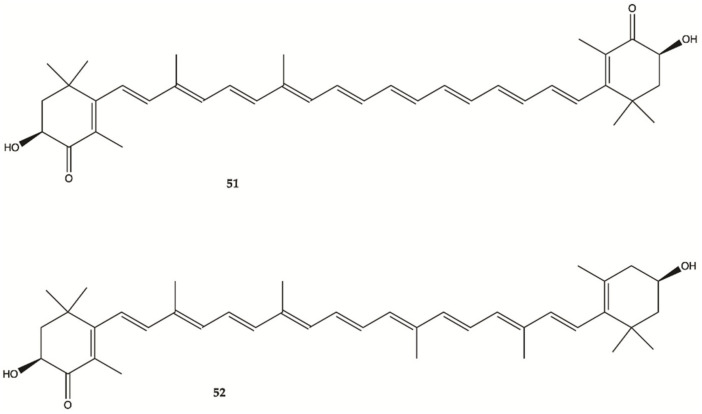
Astaxanthin (**51**) Adonixanthin (**52**) isolated from a red pigment [57].

Astaxanthine (**51**, Figure 18) is a red pigment found in crustaceans and fish that occurs naturally in shrimp, crab and salmon, and is also synthesized in *Haematococcus pluvialis*. Adonixanthin (**52**) is an intermediate product of astaxanthin (Figure 19) [57]. A cell viability assay was performed using the murine GBM cell lines GL261 and human GBM cell line U251MG cells. In GL261 cells, compounds **51** and **52** suppressed cell viability at concentration of more than 5 and 0.1 μM. In U251MG cells, compounds **51** and **52** suppressed cell viability at concentrations of more than 1 and 0.1 μM.

These two compounds inhibited both cell proliferation and migration in human and mouse glioma cells. Both were found to reduce the expression of phopharylated ERK1/2 and phosphorylated Akt [57].

## 4. ADME Properties

Effective treatment for glioma tumors is limited by the presence of the BBB. The high molecular weight of most of these marine natural products may limit BBB permeability. In this direction, we have performed in silico predictions of some pharmacokinetic parameters of the nearly fifty marine compounds presented in this review (Table 1). The development of drugs involves the assessment of absorption, distribution, metabolism and excretion (ADME), parameters that must be evaluated as early as possible that process. In this context, computational models are very good approaches, to provide access to a set of rapid, yet robust predictive models, for physical-chemical, pharmacokinetic, pharmacokinetic properties and compatibility with medicinal chemistry requirements [58]. To complement the information disclosed, the pharmacokinetic parameters were evaluated in this document using the SWISSADME platform [59].

**Table 1:** Pharmacokinetic parameters of marine compounds that are described in this review.

Compounds	Molecular Weight (g/mol)	LogP	H-Bond Acceptors-Donors 10-5	Lipinsky Rules	BBB Permeability
**1**	298.25	1.32	6-3	Yes	No
**2**	304.29	2.01	6-2	Yes	No
**3**	329.35	1.65	6-4	Yes	No
**4**	228.25	1.25	4-1	Yes	No
**5**	254.33	2.70	3-0	Yes	Yes
**6**	168.19	1.76	2-0	Yes	No
**7**	299.29	3.61	0-0	No	No
**8**	372.28	1.78	9-6	No	No
**9**	242.25	0.58	7-3	Yes	No
**10**	644.92	6.73	5-2	No	No
**11**	610.78	4.43	10-5	No	No
**12**	604.81	5.00	8-3	No	No
**13**	620.81	4.74	9-4	No	No
**14**	674.90	5.89	9-2	No	No
**15**	1243.45	5.80	18-5	No	No
**16**	1258.44	−0.33	19-5	No	No
**17**	1259.45	5.14	19-6	No	No
**18**	748.77	4.27	14-5	No	No
**19**	734.74	4.23	14-6	No	No
**20**	297.35	1.55	5-3	Yes	No
**21**	297.35	1.53	5-3	Yes	No
**22**	298.29	2.26	5-3	Yes	No
**23**	769.92	0.00	11-4	No	No
**24**	579.64	0.00	11-0	No	No
**25**	889.03	1.43	10-1	No	No
**26**	436.6	4.46	2-2	No	No
**27**	464.73	4.49	3-1	No	Yes
**28**	426.72	4.85	2-2	No	No
**29**	338.48	3.83	4-1	Yes	Yes
**30**	360.44	2.91	5-1	Yes	No
**31**	360.44	2.72	5-1	Yes	No
**32**	345.43	2.15	4-2	Yes	No
**33**	401.54	3.51	4-2	Yes	No
**34**	415.57	3.67	4-2	Yes	No
**35**	449.58	3.67	4-2	Yes	No
**36**	392.49	2.45	6-3	Yes	No
**37**	376.49	2.48	5-2	Yes	No
**38**	360.49	3.06	4-1	Yes	Yes
**39**	315.49	3.88	4-4	Yes	No
**40**	376.49	3.30	5-0	Yes	Yes
**41**	378.50	3.31	5-1	Yes	Yes
**42**	516.62	4.29	8-2	No	No
**43**	318.45	3.31	3-1	Yes	Yes
**44**	332.48	3.63	3-0	Yes	Yes
**45**	330.46	3.00	3-0	Yes	Yes
**46**	374.51	4.07	4-0	No	Yes
**47**	517.66	3.41	5-2	No	No
**48**	925.99	0.00	19-6	No	No
**49**	1221.30	4.52	20-7	No	No
**50**	882.98	0.00	17-6	No	No
**51**	568.79	6.14	4-2	No	No
**52**	582.85	6.83	3-2	No	No
**TMZ**	194.15	1.29	5-1	Yes	No

Based on the analyses of Table 1, it may be concluded that more than half of the isolated marine compounds (**1**–**6**, **9**, **20**–**22**, **29**–**41**, **43**–**46**) follow Lipinski rules [60], but only ten (**5**, **27**, **29**, **38**, **40**, **41**, **43**–**46**) are predicted to have high BBB permeability.

## 5. Nanotechnology to Improve Anti-Glioblastoma Drugs

Conventional treatments, normally, do not guarantee the necessary distribution of the drug to the tumor site, therefore hydrophilic drugs and/or low molecular weight drugs generally do not accumulate until reaching therapeutic levels in cancerous tissues, being categorized by a small circulation half-life [61]. Good drug therapy is not just related only to the molecule, but also to the delivery way [62]. Development of glioma-specific nanoparticles (NPs) has been an area of intense research over the past years. These drug delivery systems (DDS) have the capacity to be loaded with therapeutic agents and functionalized with different ligands (e.g., transferrin, chlorotoxin (CTX), hyaluronic acid) that allow targeting and crossing the BBB [63,64]. Thus, in this strategy, the ability to cross the BBB does not depend on the structure of the drug, but on the physicochemical properties of the NP, which can be improved. NP play an important role in the release of drugs through the BBB by passive diffusion, which results in a high concentration gradient between the blood and the brain and have the ability to diffuse in the vasculature with leakage of tumor tissues due to the increase in permeability and retention effect [64,65]. The deficient lymphatic drainage allows the NP to reach concentrations much higher than their concentrations in plasma [66]. NP designed for drug delivery to the brain needs to meet certain essential requirements to be more effective, such as reduced toxicity and biodegradability, prolonged circulation time, no aggregation in the blood, good encapsulation efficiency and the ability to cross the BBB [61].

Different types of nanotransporters were already applied to improve permeability and efficacy of TMZ or other innovative drugs on glioma treatment [67] as follows.

TMZ was successfully encapsulated with different functionalized NPs such as: transferrin-functionalized liposome loaded with TMZ and the bromodomain inhibitor JQ1 [68], biotin-functionalized chitosan conjugated with TMZ and surface-bound CTX ligand [69], poly(lactide-co-glycolide) (PLGA)-NP- monoclonal antibody functionalized with an OX26 mAbs [70], mesopore silica nanoparticles [71], gold-coated nanofibers [72] and magnetite NP where TMZ was loaded and incorporated into the synthesized polyurethane based on poly (ε-caprolactone diol) [73]. DOX was also tested with different NPs for glioma such as: magnetic silica NPs- functionalized with poly transferrin (D, L-lactic-co-glycolic acid) [74], biocompatible magnetic iron oxide NP stabilized with trimethoxysilylpropyl-ethylenediamine triacetic acid [75], polymeric micelle obtained from aspartic acid from poly (ethylene glycol)-b-poly (aspartic acid) block copolymer [76] and a surface-modified liposomal formulation with proteolytically stable peptides, CDX and c(RGDyK) [77]. Paclitaxel was encapsulated in an NP composed of PLGA [78] and in micelles-loaded with transferrin [79]. The anticancer efficacy of Asiatic acid (a pentacyclic triterpene, that is one of the active components of a plant, Centella Asiatica) was loaded into solid lipidic NP [80]. A potent anti-glioma synthetic xanthone derivative was synthesized and proliposomes and liposomes were developed as drug carriers [81].

It may be suggested that some of these nanotransporters may be successfully applied to improve the permeability of some of these promising anti-glioma marine compounds.

## 6. Conclusions

**Figure 20:**
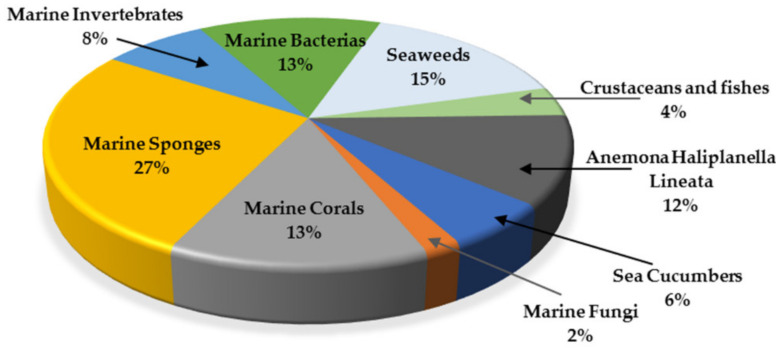
Marine organisms as sources of anti-glioma compounds.

**Figure 21:**
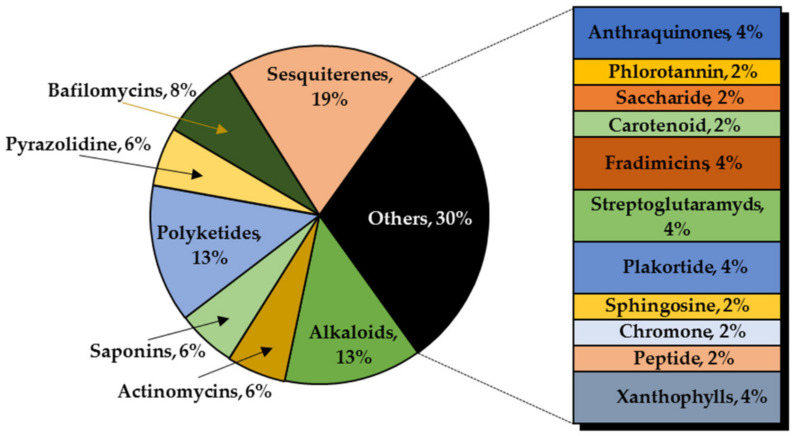
Most significant chemical classes of anti-glioma compounds.

This review summarized the compounds from marine sources found in the last decade with activity against the growth of glioma cells. Nearly fifty compounds were isolated from anemone, brown, red and green seaweeds, invertebrates, sponges, corals, fungi and crustaceos. It was possible to observe that most compounds were isolated from marine sponges (27%), followed by seaweeds (15%) and marine bacteria and marine corals (both with 13%) (Figure 20). A high chemical diversity was found: anthraquinones, chromones, peptides, pyrazolidines, sesquiterpenes, tanines, saccharides, carotenoids, Bafilomycins, Actinomycins, Fradimicins, Streptoglutarimides, alkaloids and saponins. From these, sesquiterpenes (19%), alkaloids (13%) and polyketides (13%) were the most commonly described compounds with antiproliferative activity (Figure 21).

The majority of studies have only conducted as preliminary on drug discovery processes involving in vitro screening on glioma cell lines, including, T98G, U87MG, SHG44, U251MG, U373MG, C6 among others. None have already reached advanced stages of preclinical development with proven efficacy in vivo. However, the mechanisms of action of some compounds have been elucidated, including: (i) the decrease of levels of expression of various metabolic enzymes overexpressed in the glioma, namely glycolytic enzymes (HK2, PFKFB3, PKM2 and LDH5) by compounds **1**, **2**, **15** and **48** and enzymes involved in glutaminolysis (GLS) by compounds **15** and **48**, and in lipogenesis (FASN) by compound **15**; (ii) inhibition of the AKT pathway, from which proteins are highly expressed in tumors with a worse prognosis, by compound **7**; (iii) inhibition of caspase 3/7 activity and increase of LDH activity, leading to apoptosis by compound **22**; (iv) inactivation of EGFR and the PI3K-AKT signaling pathway by compounds **23**–**25**, leading to cell death; (v) and interruption of the G2-M cell cycle and mitochondrial apoptosis by compound **40**.

From the nearly fifty marine compounds, only ten (**5**, **27**, **29**, **38**, **40**, **41**, **43**–**46**) have potential to cross BBB, being the pyrazolidene **5**, isolated from an anemone, the most potent compound (IC_50_ = 0.5 to 3.0 μM) followed by the plakortide **29** (IC_50_ = 4.0 μM) and the polyketide **38** (IC_50_ = 4.1 μM), both isolated from sponges and by the polyketide **40** (IC_50_ = 5.1 to 6.9 μM), isolated from corals.

Due to numerous advantages of the above-mentioned DDS, they can help decreasing toxicity and providing greater BBB permeability and effectiveness within the brain. It can be expected that some of the most promising compounds derived from marine sources can be applied in the future in conjunction with some of the new DDS. On the other hand, some of the presented marine compounds may work as models for the synthesis of simplified analogs in a near future.

## Data Availability

Not applicable.
